# Evaluating the feasibility, acceptability and pre testing the impact of a self-management and tele monitoring program for chronic obstructive pulmonary disease patients in Lebanon: Protocol for a feasibility study

**DOI:** 10.1097/MD.0000000000019021

**Published:** 2020-02-07

**Authors:** Rita Georges Nohra, Hala Sacre, Pascal Salameh, Monique Rothan-Tondeur

**Affiliations:** a University Paris 13, Sorbonne Paris Cite, Nursing Sciences Research chair, Laboratory Educations and Health Practices (LEPS), (EA 3412), UFR SMBH, F-93017, Bobigny, France; b Drug Information Center, Order of Pharmacists of Lebanon; c Lebanese University; Epidemiology and Biostatistics, Beirut, Lebanon; d AP HP, Nursing Sciences Research Chair Paris, France.

**Keywords:** COPD, Lebanon, quality of life, self-management, telehealth

## Abstract

**Background:**

Chronic obstructive pulmonary disease (COPD) has a significant impact on quality of life and is costly to the health care system. It has been demonstrated that a self-management program improves quality of life, but programs are not universally available and telehealth interventions can provide home-based support, but have mixed results.^[1]^

**Aim:**

The aims of this study are to (1) assess the feasibility and acceptability of a 6 weeks’ educational program related to self-management with remote monitoring for Lebanese COPD patients; (2) pre-test its impact on quality of life, emergency visits, and rate of rehospitalization, and (3) to make recommendations for a future randomized trial.

**Methods:**

Validated questionnaires will be adapted to meet the context of our study in terms of acceptability, adoption, adequacy, fidelity, cost, and coverage. The impact of this program on quality of life will be measured with the COPD assessment test (CAT) and the COPD clinical questionnaire (CCQ), and the Hospital Anxiety and Depression (HAD) scale will be used to measure anxiety. All measures will be delivered pre- and post-intervention. To evaluate the impact of our program on the rate of hospitalization and emergency visits, the number of hospitalizations and emergency room visits during the year preceding the intervention will be collected from the hospital register of each participant.

**Discussion:**

This study is the first to evaluate the application of telehealth to optimize COPD management in Lebanon. The results of this study will provide evidence regarding the efficacy and feasibility of this approach for Lebanese patients with moderate to severe COPD.

## 1 Introduction

Chronic obstructive pulmonary disease (COPD) is a progressive lung disease causing breathlessness, cough, fatigue, reduced exercise capacity, and frequent infections, with high societal burden.^[2]^ Medication optimizes airway function and reduces symptoms, but cannot address the psychosocial impact of the disease, including anxiety and depression,^[3]^ social isolation, and loss of independence and self-esteem.^[4]^

COPD is a major cause of morbidity and mortality worldwide. According to the Global Burden of Disease (GBD), COPD rose from the eighth to the fifth leading cause of GBD from 1990 to 2013.^[5]^ In 2013, COPD was the fourth leading cause of death globally, and it is predicted to become the third leading cause by 2020.^[6]^ It is the only major disease among the top 10 with a steadily increasing prevalence,^[7]^ and studies estimate that one in four persons will develop the disease in their lifetime.^[8]^ In 2010, a national study conducted in Lebanon revealed a proportion of 9.7% of COPD cases according to the GOLD definition, 80% of which were undiagnosed.^[9]^

Severe acute exacerbations COPD are associated with high rates of rehospitalization,^[10]^ worsened symptom severity,^[11]^ decreased exercise tolerance and physical activity,^[12–16]^ and negative impact on quality of life^[12]^ and mental health.^[13]^ In addition, care gaps in areas such as pharmacotherapy, inhalation technique, and knowledge of the disease are prevalent.^[17]^ Therefore, because of the burden of COPD to the health care system, the focus of its treatment has been shifting gradually from acute emergency care to a care that emphasizes self-management and maintenance.^[18]^

Self-management support interventions in COPD have been shown to improve health-related quality of life, exercise capacity, and self-efficacy^[19]^ and reduce COPD-related hospital admissions.^[20]^ As for telehealth approaches for COPD patients, several studies have reported improved knowledge and awareness of symptoms and health status.^[21,22]^ However, other studies have mixed results for self-efficacy.^[23]^ These controversies may be caused by the heterogeneity of the interventions.^[1]^

To date, in Lebanon, there are no structures for the education and care of patients with COPD. When a person with COPD is admitted to the hospital, doctors and nurses focus on their immediate problem and not on the chronicity of the disease, their overall health, or quality of life; they tend to talk to the patient about their illness rather than teach them how to manage it on a daily basis, to avoid the risk of exacerbation. Hence the need for a self-management program adapted to the Lebanese context.

The Neith program aims to study the impact of a nursing consultation and remote monitoring on the skills, knowledge, and quality of life of Lebanese patients with COPD and consists of four studies. The first study was a scoping review and showed that 4 components are essential in a self-management program for patients with COPD: initiation to the self-management program, educational sessions, support methods, and monitoring methods. The second one was a phenomenological qualitative study of 50 semi-directive interviews with Lebanese COPD patients was conducted to explore the experience of COPD patients with their disease in Lebanon, and showed that family support was a key factor in managing the disease. This third study will suggest recommendations that will lead to a fourth randomized study.

Thus, the aims of the present study are to

(1) explore the feasibility and acceptability of a remote monitoring and a self-management program for Lebanese patients with COPD,
(2) pretest the impact of this program on the quality of life, emergency visits and rehospitalization rates for this population, and
(3) make recommendations for a future randomized study.

This study will also potentially offer a framework in which COPD care can be managed in Lebanon. The present manuscript describes the protocol of the study.

## 2 Methods

### 2.1 Design

This study aims to evaluate the feasibility, acceptability and pre-test the impact of a nursing consultation and tele-monitoring in Lebanon. It will be conducted at the Hôtel-Dieu de France (HDF) hospital, one of the largest university hospitals in Beirut, and will use quantitative and qualitative methods in pre- and post-intervention to collect data over a period of 3 months with a single group intervention. Data will be collected by 2 researchers. A consent form will be signed by each participant before the intervention. Patients will be informed about the purpose of the study, the course of the intervention, and the freedom to withdraw from the study at any time. Their consent for the video recording will also be taken.

### 2.2 Ethical aspects

This study has been approved by HDF Ethics Committee record CEHDF 1519, November 4, 2019. It will be conducted in accordance with the protocol. The rules of confidentiality will be respected.

### 2.3 Setting and participants

An accidental empirical sample will be recruited based on the judgment of the pulmonologists at HDF hospital and on a voluntary basis from 30 patients with moderate to severe COPD.

To be considered eligible, in addition to signing the informed consent, patient must be adult (18 years of age or older), male or female, outpatient, with moderate (Gold 2) or severe (Gold 3) COPD, cognitively capable, with an adequate health status to participate in the study according to the clinical consensus between nurses and physicians.

Patients will be excluded if they are diagnosed with lung cancer or if they have cognitive problems related to memory loss or speech disorders that would not allow a constructive exchange.

### 2.4 Intervention development

**Figure 1:**
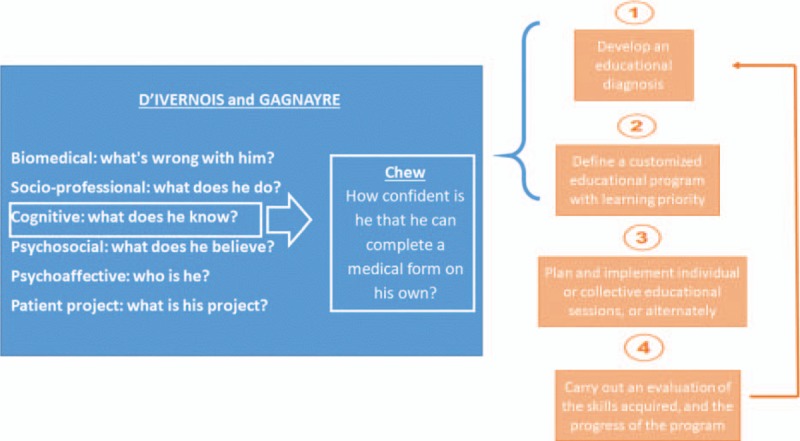
Framework of the intervention.

**Figure 2:**
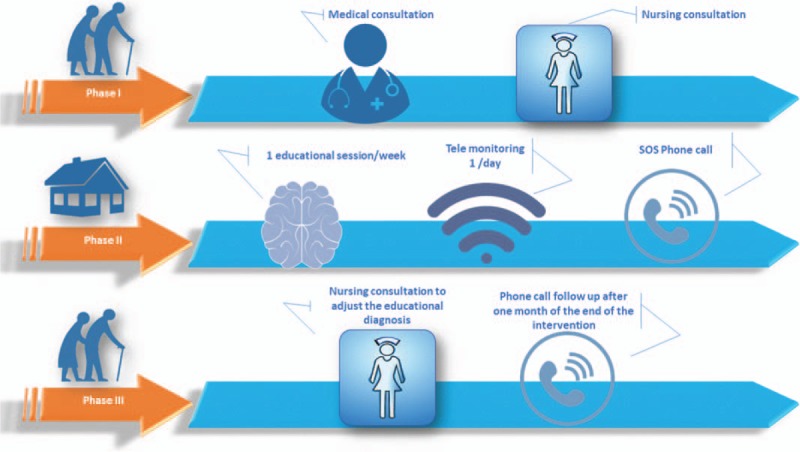
Progress of the intervention.

This intervention is based on the recommendations of the High Authority of Health (HAS) in France to plan a therapeutic education program (ETP),^[23]^ and on the D’Ivernois and Gagnayre model to build the educational diagnosis and plan the educational sessions.^[24]^ Health literacy will be assessed by the question proposed by Chew (Fig. 1).^[25]^ Our intervention will be based on the following framework and will consist of 3 phases (Fig. 2):

**Phase I:** At the end of a medical consultation, the doctor will offer the patient the opportunity to benefit from a nursing consultation and remote monitoring to better manage his COPD. A nursing consultation will be scheduled during which the nurse will explain the procedure, obtain the written consent of the patient, and develop an educational diagnosis with the participation of the patient. This step consists of a motivational interview that constitutes a guided, person-centered approach aiming at making patients aware of their behavior and consider actions to change it if it adversely affects their health. The nurse will explain how the patient should use the oximeter to measure daily the oxygen saturation (SaO2) and pulse, and how the information collected will be transmitted. An appointment will then be made for a first meeting at the patient's home.

**Phase II:** During the next 6 weeks the patient will receive one visit per week from the nurse to encourage him/her to self-assess each of his/her risk factors so that he/she becomes aware that his/her illness is not inevitable and that he/she can be involved in a behavioral change to reduce the risk of exacerbations. During these 6 weeks, a telephone number will be available to the patient in case of emergency and for any additional information.

**Phase III:** Following the 6 educational sessions delivered by the nurse, a final consultation will be scheduled at the nurse office to evaluate the patient's achievements and modify his/her educational assessment. A telephone follow-up will be conducted after one month to assess the maintenance of learning.

### 2.5 Outcome measures

Based on Proctor's lists of outcome variables that can assess program implementation,^[26]^ this study will evaluate the acceptability, adoption, adequacy, fidelity, cost, and coverage of the intervention, using various tools: the “Pre-Referral intervention team inventory” will be adapted to study the acceptability of the intervention,^[27]^ the “adoption of information technology innovation” scale for adoption,^[28]^ the “Parenting strategies questionnaire” for adequacy,^[29]^ the “Utilization and Cost Questionnaire” for cost,^[30]^ and the “levels of institutionalization” scale for coverage.^[31]^ With regard to fidelity, no evidence-based instrument could be identified; thus, the fidelity will be assessed by comparing the protocol of the intervention with what will be done in the field, the education sessions will be videotaped and a second researcher will be invited to evaluate the fidelity.

To evaluate the impact of the intervention, many pre-tests will be performed before starting it: the COPD assessment test (CAT)^[32]^ to evaluate the quality of life of participants, the COPD clinical questionnaire (CCQ)^[33]^ to evaluate their respiratory health status, and the “Hospital Anxiety and Depression” (HAD) scale to measure the anxiety.^[34]^ Skills and expertise of the participants will be assessed using questions created for each session and comparing the pre- and post-intervention educational diagnosis. The hospital register of each participant will be consulted to collect and evaluate the number of hospitalizations and emergency room visits during the year preceding the intervention and during the intervention.

All these measurements will be grouped into evaluation sheets. There will be sheets to collect data from participants, and others to collect data from researchers so that the program can then be evaluated.

### 2.6 Data collection and follow-up

**Figure 3:**
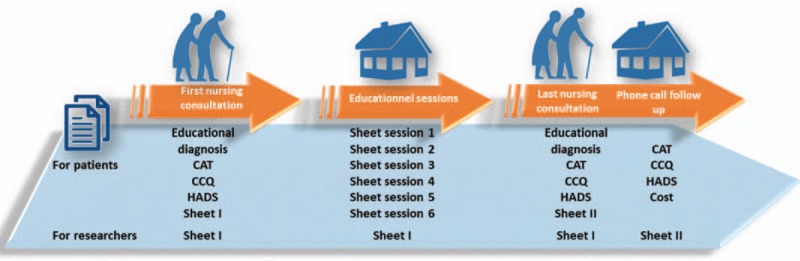
Data collection and follow-up.

Figures 2 and 3 summarize all the phases and the different evaluations performed at each level.

#### 2.6.1 First phase

In the first phase of our intervention the quality of life of participants, their respiratory health status, and their level of anxiety will be measured using specific tools. Participants’ skills and expertise will be evaluated by an educational diagnosis. The hospital register of each participant will be consulted to collect the number of hospitalizations and emergency room visits during the year preceding the intervention. Patients will be asked, at the end of the session, to complete sheet 1 of the evaluation that includes 4 parts about their perception of the acceptability, adequacy, adoption, and cost of the program.

A 2-part sheet about the cost and fidelity will be completed by 2 researchers. The cost part will be completed by the principal investigator after each nursing consultation. The fidelity part will be completed by a second researcher who will be invited to participate in the evaluation of the program. All sessions will be video or audio recorded to facilitate this task (Fig. 3).

#### 2.6.2 Second phase

At this phase 6 educational sessions will be conducted at the patients’ place. Before each session, patients will be required to complete a 5-question questionnaire, to assess their knowledge of the topic. At the end of each session, patients will have to fill the same questionnaire again to evaluate their short-term learning. They will also be required to evaluate the acceptability and cost of the session. An open-ended question is added to explore the participant's perception of the education session. After each session, researchers will complete their evaluation sheet.

#### 2.6.3 Third phase

During this last session that takes place at the nurse's office, quality of life of participants, their respiratory health status, and their level of anxiety will be reevaluated. A second educational diagnosis will be performed and compared to the first one. At the end of the session, patients will be asked to complete sheet 2 of the evaluation that includes 4 parts about their perception of the acceptability, adequacy, adoption, and cost of the program.

#### 2.6.4 Phone call follow up

One month after the last intervention, using the phone, a third evaluation of the quality of life of participants, their respiratory health status, and their level of anxiety will be performed by the principal investigator who will also identify the rate of re-hospitalization and emergency visits for each participant in the month that follows the end of the intervention. The principal researcher will then complete the cost part of the 2-part sheet.

Researchers will complete the first part concerning the cost, which will be completed by the principal investigator. A second researcher will complete the fidelity part in addition to the Loln questionnaire that assesses coverage.

### 2.7 Sample size calculation

Since this is a feasibility study with an intervention that has never been tested before on the Lebanese population, no formal calculation of the sample size will be performed, and 30 patients with moderate to severe COPD will be recruited, based on Hertzog's recommendations: a sample of 10 to 40 people is sufficient for a pilot study using a single group to estimate sample size for a future trial.^[35]^

### 2.8 Analyses

To determine the feasibility of this research program, 6 variables will be selected: acceptability, adoption, adequacy, fidelity, cost and coverage, with 2 types of participants: COPD patients and researchers. Patients will assess acceptability, adoption, adequacy and cost, while researchers will assess the fidelity, cost and coverage of the program.

Analyses will be performed on SPSS version 25 (IBM SPSS Statistics for Windows). Student *t* test will be used to compare normally distributed continuous variables between 2 groups, ANOVA test between 3 groups or more, and Chi-square for binary and/or categorical variables. Open ended questions will be analyzed narratively.

### 2.9 Potential limitation and bias

#### 2.9.1 Selection bias

It is defined as the representation of the results of a sample that is not representative of the entire population concerned. Our study might be subject to this bias since only voluntary, and therefore motivated patients will be included in our study. Documenting the reasons for non-participation is a planned strategy to limit this bias.

#### 2.9.2 Attrition bias

The attrition bias is defined as the observation of systematic differences between groups due to premature withdrawal from the study leading to incomplete results data. To reduce the effect of this bias, the “intention to treat” analysis will be adopted, which means that the analysis will take into account all included patients who have completed the study or not.

#### 2.9.3 Social desirability bias (Hawthorne effect)

It is defined by the observed positive effect that is not related to the intervention but simply due to the fact that this group feels observed, hence the modification of its behavior. This limitation can be observed in our study since the protocol design does not include a blind group. A telephone follow-up is therefore considered 1 month after the intervention to evaluate the maintenance of the acquired skills.

#### 2.9.4 Confirmation bias

This tendency to favor information that confirms preconceived ideas can be observed in our study in the need to judge whether the intervention was actually received and faithful to the original protocol. To reduce the effect of this bias, a second judge will be asked to evaluate this step.

## 3 Discussion

As the prevalence of COPD continues to increase, so does its cost to the healthcare system. Self-management has been shown to be an effective component of chronic care, yet the impact of tele-monitoring has not been yet established as a support for a self-management program. This proposed intervention may improve the quality of life and reduce emergency visits and re-hospitalization rates of Lebanese patients with COPD; it may also be extended to COPD patients in any developing country with no specific educational program for this population.

Results of this innovative study in Lebanon will establish feasibility and provide preliminary evidence on the efficacy of this intervention, and could potentially influence clinical practice and health policy in Lebanon.

### 3.1 Trial status

This protocol is registered on clinicalTrials.gov 11 December 2019, NCT04196699.

At the time of manuscript submission, participant will be recruited.

## Acknowledgments

Our acknowledgments to Docteur Zeina Aoun, Georges Dabar, Moussa Riachi, Georges Khayat, Ihab Ibrahim for providing a list of their patients to be include in this study.

## Author contributions

**Conceptualization:** Rita Georges Nohra, Monique Rothan-Tondeur.

**Supervision:** Monique Rothan-Tondeur.

**Validation:** Pascale Salameh, Monique Rothan-Tondeur.

**Visualization:** Pascale Salameh, Monique Rothan-Tondeur.

**Writing – review & editing:** Rita Georges Nohra, Hala Sacre.

Rita Georges Nohra orcid: 0000-0002-8020-3224.
